# Bioinformatic Prediction of Activation States in Molecular Network Pathways of Eukaryotic Initiation Factor 2 (EIF2) Signaling and Coronavirus Pathogenesis

**DOI:** 10.3390/ijms27031525

**Published:** 2026-02-04

**Authors:** Shihori Tanabe, Sabina Quader, Ryuichi Ono, Hiroyoshi Y. Tanaka, Horacio Cabral

**Affiliations:** 1 Division of Risk Assessment, Center for Biological Safety and Research, National Institute of Health Sciences, Kawasaki 210-9501, Japan; 2 Innovation Centre of NanoMedicine (iCONM), Kawasaki Institute of Industrial Promotion, Kawasaki 210-0821, Japan; 3 Division of Cellular and Molecular Toxicology, Center for Biological Safety and Research, National Institute of Health Sciences, Kawasaki 210-9501, Japan; onoryu@nihs.go.jp; 4 Department of Pharmaceutical Biomedicine, Graduate School of Medicine, Dentistry and Pharmaceutical Sciences, Okayama University, Okayama 700-8530, Japan; hiroyoshi.y.tanaka@okayama-u.ac.jp; 5 Department of Bioengineering, Graduate School of Engineering, The University of Tokyo, Tokyo 113-0033, Japan; horacio@g.ecc.u-tokyo.ac.jp

**Keywords:** coronavirus, EIF2 signaling, microRNA, molecular pathway, network activation

## Abstract

Eukaryotic initiation factor 2 (EIF2) signaling plays a crucial role in regulating mRNA translation and initiating eukaryotic protein synthesis. Computational molecular network pathway analysis of the canonical pathways of the coronaviral infection revealed that EIF2 signaling is inactivated when the coronavirus pathogenesis pathway is activated and vice versa. Our computational analyses indicated that the coronavirus pathogenesis pathway and EIF2 signaling had inverse activation states. Computational investigation of upstream or downstream microRNA (miRNA) revealed that EIF2 signaling directly interacted with miRNAs, including let-7, miR-1292-3p (miRNAs with the seed CGCGCCC), miR-15, miR-34, miR-378, miR-493, miR-497, miR-7, miR-8, and MIRLET7. A total of 36 nodes, including 8 molecules (ATF4, BCL2, CCND1, DDIT3, EIF2A, EIF2AK3, EIF4E, and ERK1/2), 1 complex (the ribosomal 40s subunit), and 1 function (apoptosis) in the coronavirus pathogenesis pathway, overlapped with EIF2 signaling. Alterations in EIF2 signaling may play a role in the pathogenesis of coronavirus.

## 1. Introduction

Eukaryotic translation initiation factor 2 (EIF2) is a guanosine 5′-diphosphate (GDP)-binding protein with three subunits (alpha, beta, and gamma) that delivers initiator tRNA (Met-tRNAi) to 40S ribosomes in a guanosine 5′-triphosphate (GTP)-dependent manner [1]. EIF2 signaling contributes physiologically to the initiation of protein synthesis and the integrated stress response (ISR) [2]. The ISR involves a ternary complex composed of EIF2, GTP, and charged Met-tRNAi [3] and is triggered by various stress conditions that alter cellular homeostasis [2]. Sensor kinases HRI (heme-regulated inhibitor, encoded by the EIF2 alpha kinase 1 (EIF2AK1) gene), PKR (double-stranded RNA-dependent protein kinase, encoded by the EIF2AK2 gene), and PERK (PKR-like endoplasmic reticulum kinase, encoded by the EIF2AK3 gene), in addition to GCN2 (general control non-repressible 2, encoded by the EIF2AK4 gene), contain both conserved kinase domains and divergent regulatory domains that enable them to respond to different stimuli [2].

The coronavirus pathogenesis pathway is activated upon coronaviral infection. The coronavirus pathogenesis pathway involves mitogen-activated protein kinase (MAPK) signaling, interferon (IFN) signaling, transforming growth factor (TGF)-beta signaling, and nuclear factor (NF)-kappa B signaling, leading to the inhibition of innate immunity and the dysfunction of T cells [4]. Although the coronavirus pathogenesis and coronavirus replication pathways include known signaling pathways, their precise mechanisms and regulation of activity have yet to be revealed. In this study, we focused on EIF2 signaling as a hub and compared the activation states of the EIF2 signaling and the coronavirus pathogenesis pathway using the Ingenuity Pathway Analysis (IPA) computational network pathway analysis tool and gene expression databases. We investigated whether the EIF2 signaling pathway and the coronavirus pathogenesis pathway are inversely activated in public gene expression datasets, defined their integration with miRNA interactions, and conducted a cross-tissue comparison of EIF2 signaling and the coronavirus pathogenesis pathway.

## 2. Results

### 2.1. EIF2 Signaling in Severe Acute Respiratory Syndrome Coronavirus 2 (SARS-CoV-2)-Infected Induced Pluripotent Stem (iPS) Cells

**Figure 1:**
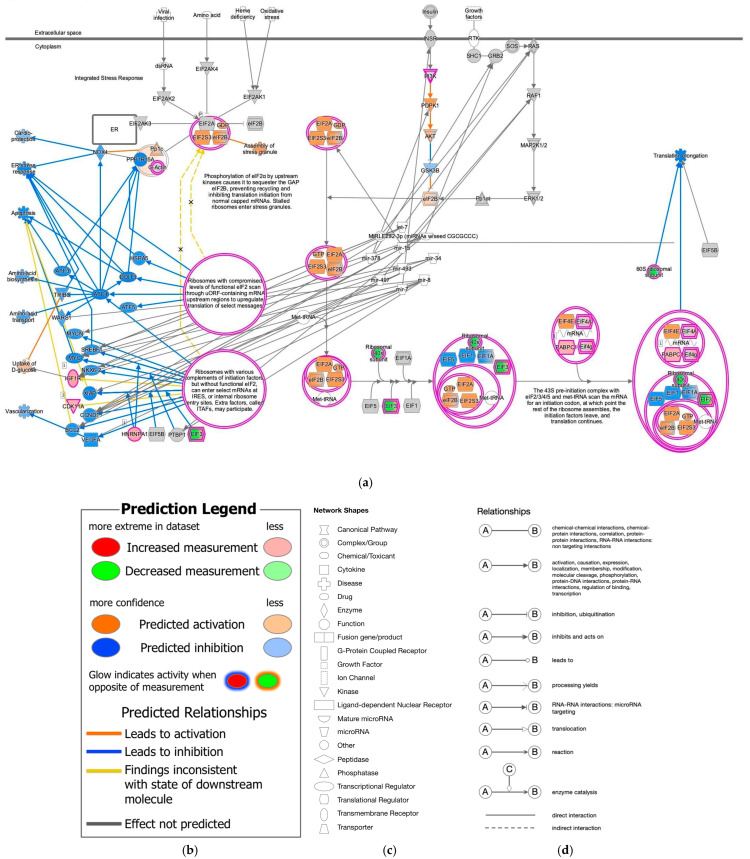
Computational pathway analysis suggests reduced EIF2 signaling activity in SARS-CoV-2-infected iPS cells. (**a**) Result for 1-normal-control-skin-infection-SARS-coronavirus-2-(SARS-CoV-2)-6408 was overlaid onto the EIF2 signaling. (**b**) The pathway’s prediction legend. Red and green coloring indicate upregulated and downregulated gene expression, respectively. Orange and blue coloring indicate predicted activation and inhibition, respectively. The intensities of the colors indicate the degree of up- or downregulation. Orange and blue lines indicate activation and inhibition, respectively. Solid and dashed lines indicate direct and indirect interaction, respectively. (**c**) The legend for the node shapes in the pathway. (**d**) The legend for the relationship lines.

During our previous study on the coronavirus pathogenesis pathway [4], we found that EIF2 signaling was inactivated in SARS-CoV-2-infected induced pluripotent stem (iPS) cells. In the computational analysis of SARS-CoV-2-infected iPS cells, the gene expression of EIF3 (complex) was decreased, and EIF1, EIF1A, and EIF5 were predicted to be inhibited (Figure 1). Translation elongation was predicted to be inhibited in SARS-CoV-2-infected iPS cells, as shown in Figure 1. EIF2A, EIF2S3, and EIF2B (complex) (eIF2B) were predicted to be activated in the EIF2 signaling of SARS-CoV-2-infected iPS cells. The source dataset of the computational analysis is specified in Table 1. The Gene Expression Omnibus (GEO) accession number of the source dataset is GSE156754 (https://www.ncbi.nlm.nih.gov/geo/query/acc.cgi?acc=GSE156754, accessed on 17 December 2025) [5].

EIF2B (complex) consists of EIF2B1, EIF2B2, EIF2B3, EIF2B4, and EIF2B5 and is a member of 43S TRANSLATION PREINITIATION (complex), 48S RIBOSOME (complex), EIF2 (complex), and MET/TRNA/EIF2 (complex). EIF2B (complex) is involved in the cachexia signaling pathway, cardiac hypertrophy signaling, cardiac hypertrophy signaling (enhanced), estrogen receptor signaling, eukaryotic translation initiation, the IFN-gamma-activated inhibitor of translation (GAIT) signaling pathway, insulin receptor signaling, the insulin secretion signaling pathway, the regulation of EIF4 and p70S6K signaling, and the stress granule signaling pathway, in addition to EIF2 signaling in the IPA canonical pathway knowledge base. Key molecules in EIF2 signaling are summarized in Table 2.

### 2.2. Activation State of EIF2 Signaling

**Figure 2:**
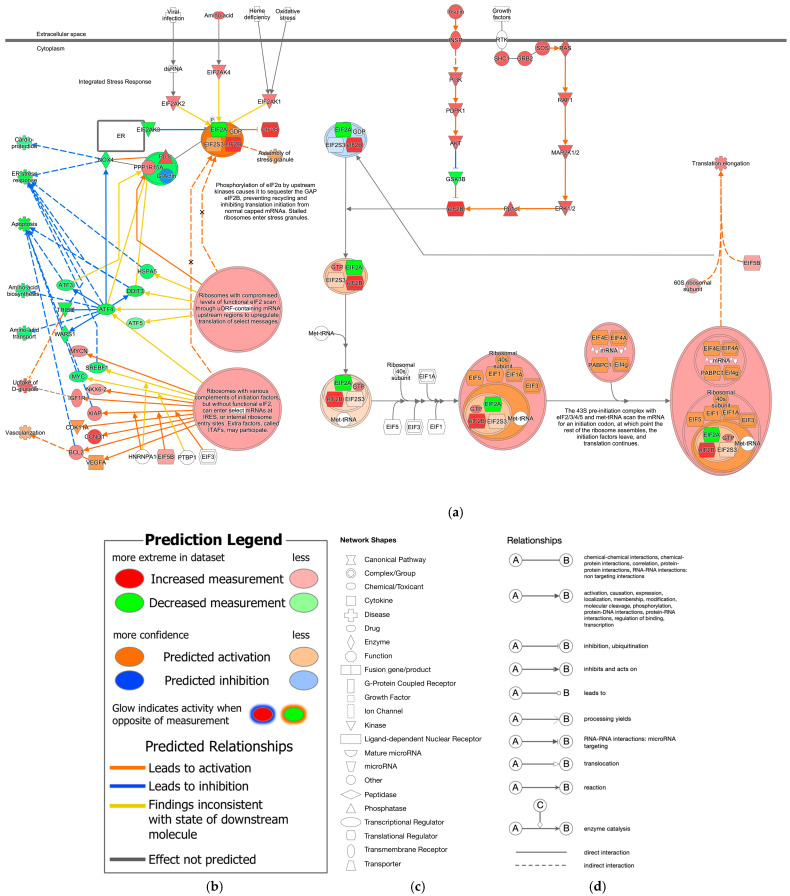
Expected activation state of EIF2 signaling in computational pathway analysis. (**a**) The expected activation state is based on the literature available in the IPA knowledge base. (**b**) The pathway’s prediction. Red and green indicate upregulated and downregulated gene expression, respectively. Orange and blue indicate predicted activation and inhibition, respectively. The intensities of the colors indicate the degree of up- or downregulation. Orange and blue lines indicate activation and inhibition, respectively. Solid and dashed lines indicate direct and indirect interaction, respectively. (**c**) The legend for the node shapes in the pathway. (**d**) The legend for the relationship lines.

EIF2 signaling and the expected activation state of EIF2 signaling in IPA are shown in Figure 2. Amino acid activates EIF2AK4, leading to the assembly of stress granules, while RAS signaling activates eIF2B, leading to the initiation of translation by the 43S preinitiation complex with EIF2/3/4/5 and Met-tRNA (Figure 2). The expected activation state of EIF2 signaling contained several types of nodes, including increased/decreased measurement and predicted activation/inhibition in computational pathway analysis.

### 2.3. Activity Plots of the Coronavirus Pathogenesis Pathway

**Figure 3:**
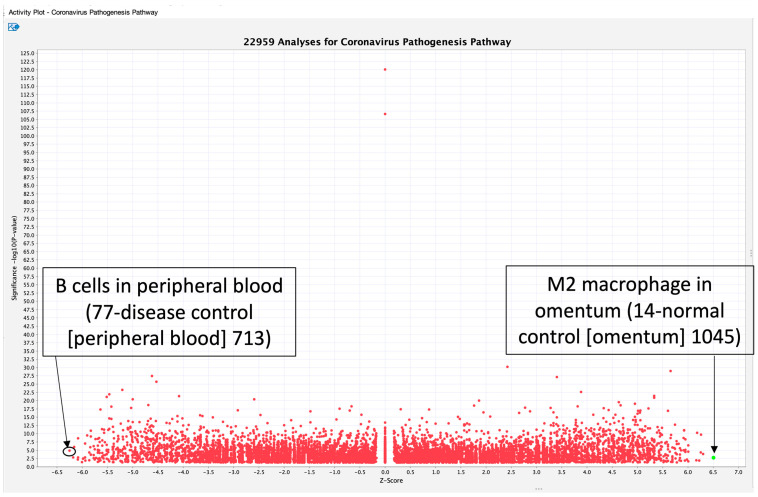
Activity plots of the coronavirus pathogenesis pathway using IPA computational analysis. A total of 22,959 analyses were identified in coronavirus pathogenesis pathway activity plots. An analysis of M2 macrophages in the omentum showed the highest activation z-score among the coronavirus pathogenesis pathway activity plots. The designation of that analysis was 14-normal control (omentum) 1045, as of October 2022. An analysis of B cells in peripheral blood showed the lowest activation z-score among the coronavirus pathogenesis pathway activity plots. The designation of that analysis name was 77-disease control (peripheral blood), as of October 2022.

To reveal the activation states of the coronavirus pathogenesis pathway under various changes in gene expression, activity plots of the coronavirus pathogenesis pathway were analyzed using the IPA database to identify data with the highest or lowest activation z-score. Activity plots of the coronavirus pathogenesis pathway are shown in Figure 3. A total of 22,959 analyses were identified in coronavirus pathogenesis pathway activity plots. The 22,959 analyses were defined using a canonical pathway search with the term “Coronavirus Pathogenesis Pathway” and by selecting the activity plot option under the optional license of IPA computational pathway analysis as of 17 October 2022. At that time, the number of gene expression data analyses relating to the coronavirus pathogenesis pathway increased. The datasets (analyses) in the IPA database under the optional license, which included the coronavirus pathogenesis pathway as an associated canonical pathway as of 17 October 2022, were computationally selected. Detailed information on datasets that have absolute z-scores greater than 3 is available in Supplementary Table S1. M2 macrophages in the omentum had the highest activation z-score, indicating that the gene expression pattern of the molecules in the dataset is similar to the pattern expected based on the available literature (the coronavirus pathogenesis pathway activity plot as of October 2022 (Figure 3)). The gene expression of M2 macrophages in the dataset was compared to that of B cells, dendritic cells, fibroblasts, mast cells, mesothelial cells, stromal cells, and T cells (Figure 3). An analysis of B cells in peripheral blood showed the lowest z-score in coronavirus pathogenesis pathway activity plots as of October 2022 (Figure 3). In the dataset, gene expression in B cells was compared to that in monocytes, neutrophils, platelets, T cells, and unassigned cells, indicating that the gene expression pattern of B cells is less similar to the pattern expected based on the available data on the coronavirus pathogenesis pathway compared to other cells in peripheral blood (Figure 3).

### 2.4. EIF2 Signaling Inhibition of the M2 Macrophage in Omentum with the Most Activated Coronavirus Pathogenesis Pathway

**Figure 4:**
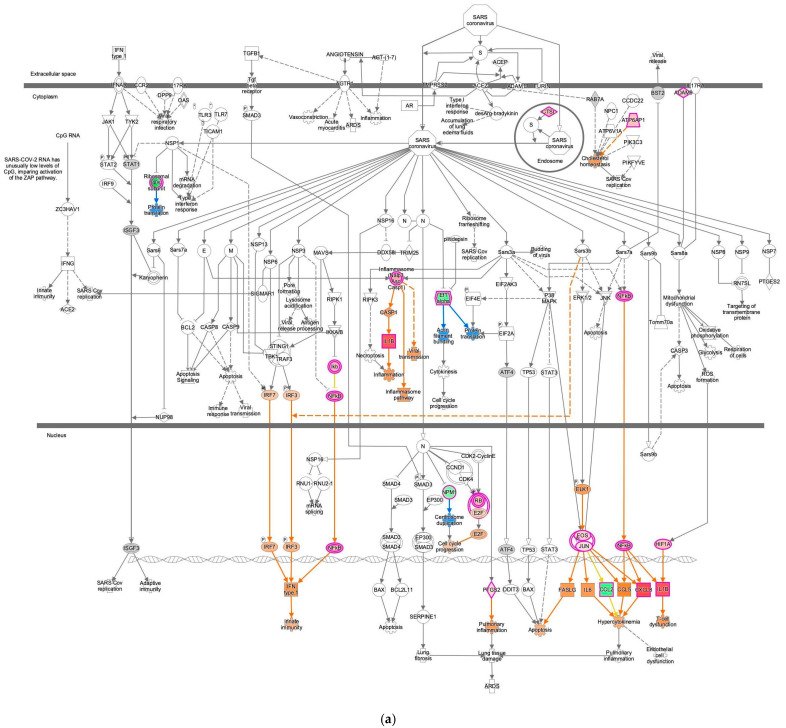

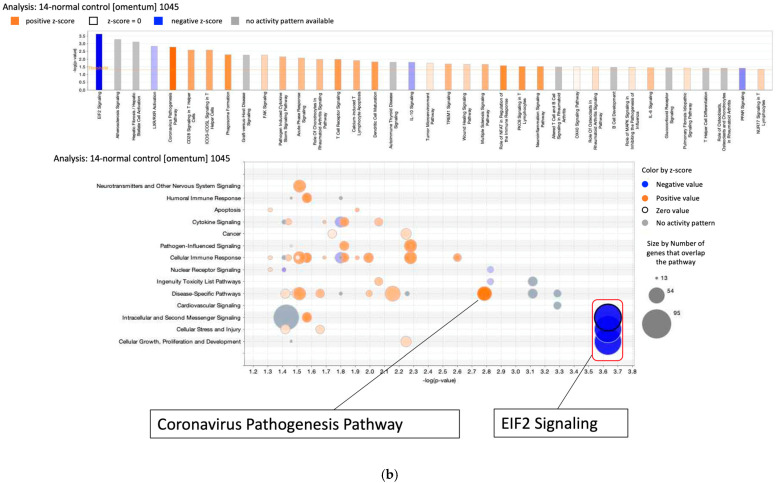
Computational pathway analysis suggests increased coronavirus pathogenesis pathway activity in M2 macrophages in the omentum. (**a**) The result for 14-normal control (omentum) 1045 was overlaid onto the coronavirus pathogenesis pathway. Red and green indicate upregulated and downregulated gene expression, respectively. Orange and blue indicate predicted activation and inhibition, respectively. The intensities of the colors indicate the degree of up- or downregulation. Orange and blue lines indicate activation and inhibition, respectively. Solid and dashed lines indicate direct and indirect interaction, respectively. (**b**) Computational pathway analysis suggests increased coronavirus pathogenesis pathway activity and reduced EIF2 signaling activity in M2 macrophages.

**Figure 5:**
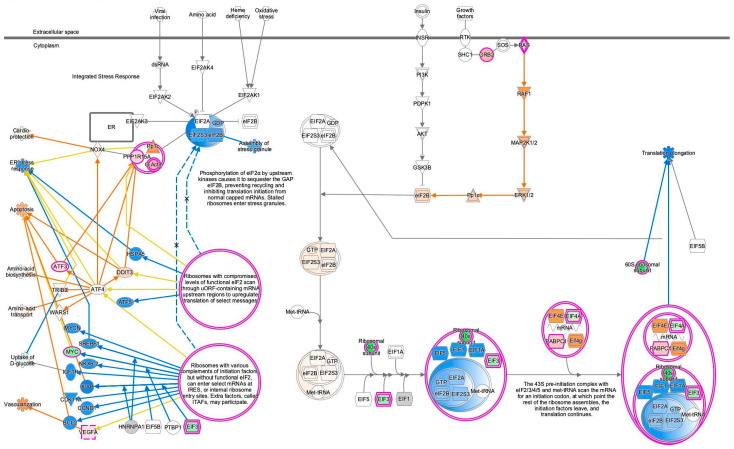
The result for 14-normal control (omentum) 1045 was overlaid onto the EIF2 signaling. Computational pathway analysis suggests reduced EIF2 signaling activity in macrophages in the omentum. Red and green indicate upregulated and downregulated gene expression, respectively. Orange and blue indicate predicted activation and inhibition, respectively. The intensities of the colors indicate the degree of up- or downregulation. Orange and blue lines indicate activation and inhibition, respectively. Solid and dashed lines indicate direct and indirect interaction, respectively.

The coronavirus pathogenesis pathway was activated in M2 macrophages in the omentum (Figure 4). The result for 14-normal control (omentum) 1045 was overlaid onto the coronavirus pathogenesis pathway, which showed activation of the coronavirus pathogenesis pathway in computational pathway analysis (Figure 4). The same analysis result was overlaid onto the EIF2 signaling pathway, which showed inactivation of the EIF2 signaling (Figure 4b and Figure 5). In the 14-normal control (omentum) 1045 analysis, the M2 macrophages were compared to other cell types, such as plasma B cells, dendritic cells, fibroblasts, mast cells, mesothelial cells, stromal cells, and T cells, in the male omentum tissue of the normal control in the IPA database. In the IPA analysis of miRNA interactions with EIF2 signaling, nine microRNAs (miRNAs) (let-7, miR-1292-3p (miRNAs w/seed CGCGCCC), miR-15, miR-34, miR-378, miR-493, miR-497, miR-7, and miR-8) and one group (MIRLET7) were identified as having direct relationships with EIF2 signaling (Table 3). The computational analysis was conducted purely using knowledge-based prediction as part of IPA; this hypothesis-generating prediction is the limitation of this study that will require validation in future investigations.

### 2.5. Enrichment Analysis of the Predicted Target Genes of microRNAs (miRNAs)

To further investigate the roles of miRNAs associated with EIF2 signaling, enrichment analysis was performed on the predicted target genes of the miRNAs. The predicted target genes of the nine miRNAs and one group indicated in Table 3 that have direct relationships with EIF2 signaling are shown in Table 4 and Supplementary Table S2.

The target genes were uploaded to the Database for Annotation, Visualization, and Integrated Discovery (DAVID), which resulted in the analysis of 528 *Homo sapiens* genes. Functional annotation clustering of the enrichment analysis showed that chronic myeloid leukemia, hepatitis B, cellular senescence, human T-cell leukemia virus 1 infection, and viral carcinogenesis were included in the top cluster with the highest enrichment scores (Table 5, Supplementary Table S3).

### 2.6. EIF2 Signaling Activation in B Cells in Peripheral Blood with the Least Activated Coronavirus Pathogenesis Pathway

**Figure 6:**
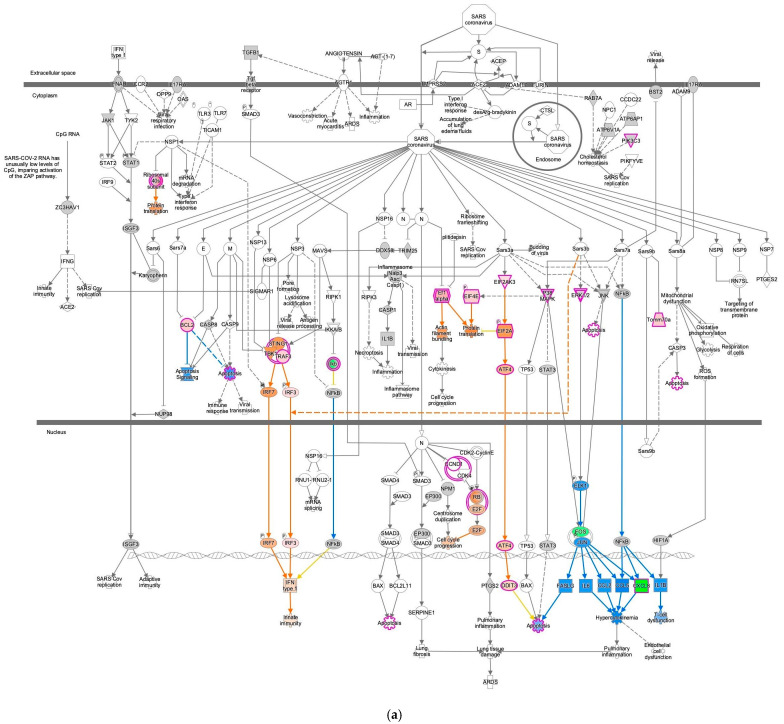

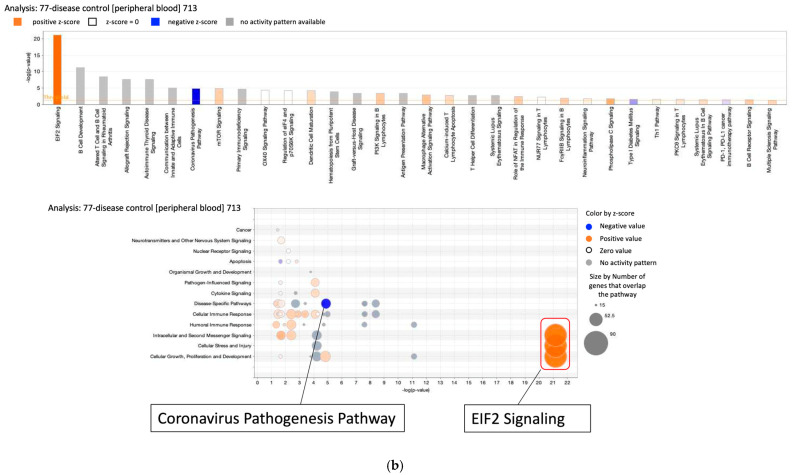
Computational pathway analysis suggests reduced coronavirus pathogenesis pathway activity in B cells in peripheral blood. (**a**) The result for 77-disease control (peripheral blood) 713 is overlaid onto the coronavirus pathogenesis pathway. Red and green indicate upregulated and downregulated gene expression, respectively. Orange and blue indicate predicted activation and inhibition, respectively. The intensities of the colors indicate the degree of up- or downregulation. Orange and blue lines indicate activation and inhibition, respectively. Solid and dashed lines indicate direct and indirect interaction, respectively. (**b**) Computational pathway analysis suggests reduced coronavirus pathogenesis pathway activity and increased EIF2 signaling activity in B cells in peripheral blood.

**Figure 7:**
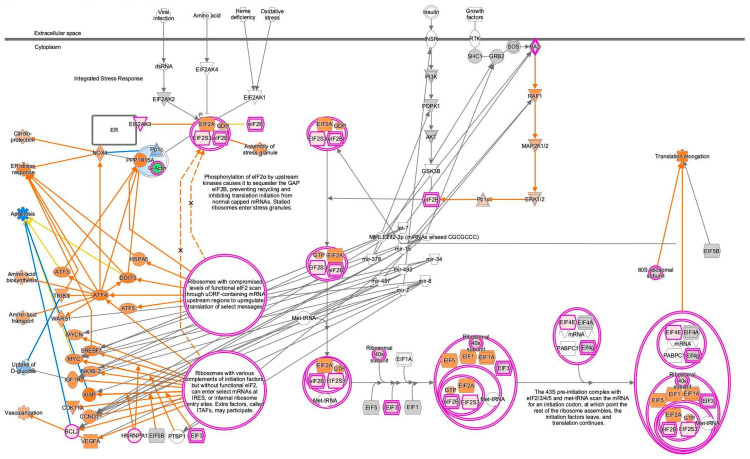
The result for 77-disease control (peripheral blood) 713 was overlaid onto EIF2 signaling. Computational pathway analysis suggests increased EIF2 signaling activity in B cells in peripheral blood. Red and green indicate upregulated and downregulated gene expression, respectively. Orange and blue indicate predicted activation and inhibition, respectively. The intensities of the colors indicate the degree of up- or downregulation. Orange and blue lines indicate activation and inhibition, respectively. Solid and dashed lines indicate direct and indirect interaction, respectively.

Computational pathway analysis suggested reduced coronavirus pathogenesis pathway activity in peripheral blood B cells (Figure 6). The result for the 77-disease control (peripheral blood) 713 analysis was overlaid onto the coronavirus pathogenesis pathway, which showed inactivation of the coronavirus pathogenesis pathway (Figure 6). The same analysis result was overlaid onto EIF2 signaling, and this showed increased EIF2 signaling activity in computational pathway analysis (Figure 6b and Figure 7). The disease state of the 77-disease control (peripheral blood) 713 analysis was normal. The gene expression data were obtained from adult female peripheral blood B cells, and other cells (monocytes, neutrophils, platelets, T cells, and unassigned cells) were used for comparison in the analysis performed using the IPA database. The data source is available in ArrayExpress with Accession number E-MTAB-9221 (https://www.ebi.ac.uk/biostudies/arrayexpress/studies/E-MTAB-9221, accessed on 18 November 2025) [6]. Detailed information of the 77-disease control (peripheral blood) 713 analysis is also presented in Supplementary Table S1.

**Figure 8:**
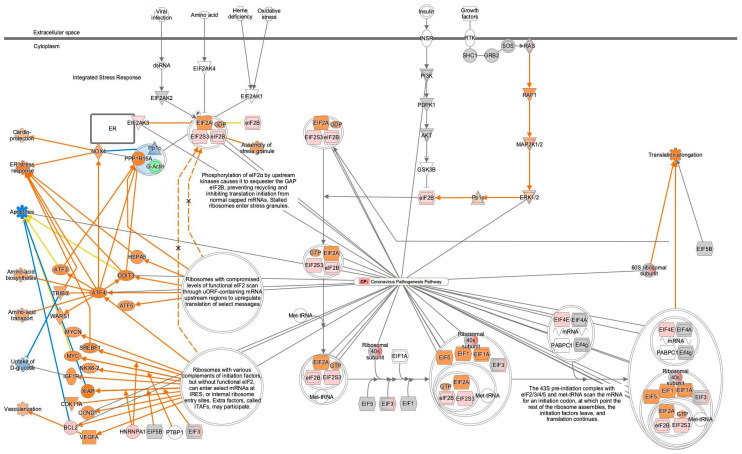
A total of 36 nodes, including 8 molecules (ATF4, BCL2, CCND1, DDIT3, EIF2A, EIF2AK3, EIF4E, ERK1/2), 1 complex (ribosomal 40s subunit), and 1 function (apoptosis) from the coronavirus pathogenesis pathway, were overlapped with EIF2 signaling. The result for 77-disease control (peripheral blood) 713 was overlaid onto EIF2 signaling. Red and green indicate upregulated and downregulated gene expression, respectively. Orange and blue indicate predicted activation and inhibition, respectively. The intensities of the colors indicate the degree of up- or downregulation. Orange and blue lines indicate activation and inhibition, respectively. Solid and dashed lines indicate direct and indirect interaction, respectively.

A total of 36 nodes, including 8 molecules (ATF4, BCL2, CCND1, DDIT3, EIF2A, EIF2AK3, EIF4E, and ERK1/2), 1 complex (the ribosomal 40s subunit), and 1 function (apoptosis) in coronavirus pathogenesis, were overlapped with EIF2 signaling (Figure 8, Table 6).

### 2.7. EIF2 Signaling in Diffuse-Type and Intestinal-Type Gastric Cancer

To investigate whether the inverse relationship between the activation states of the coronavirus pathogenesis pathway and EIF2 signaling holds in other disease states, we analyzed diffuse-type and intestinal-type gastric cancer, in which we had previously observed the differential dysregulation of the coronavirus pathogenesis pathway.

**Figure 9:**
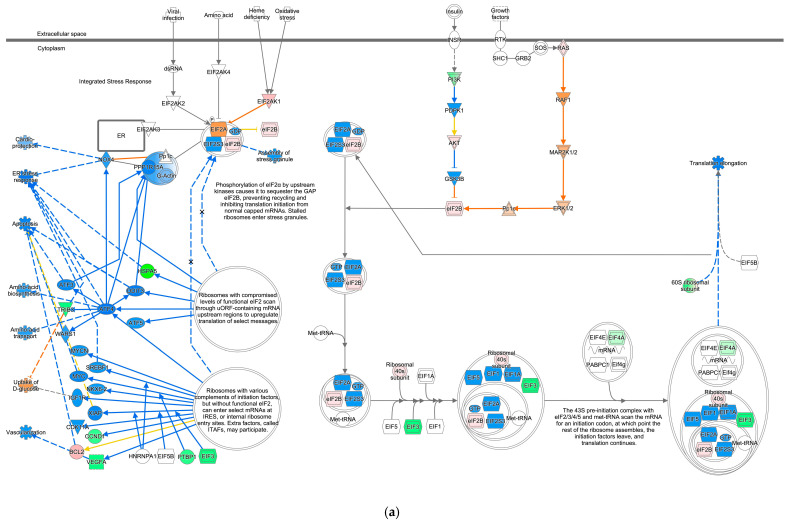

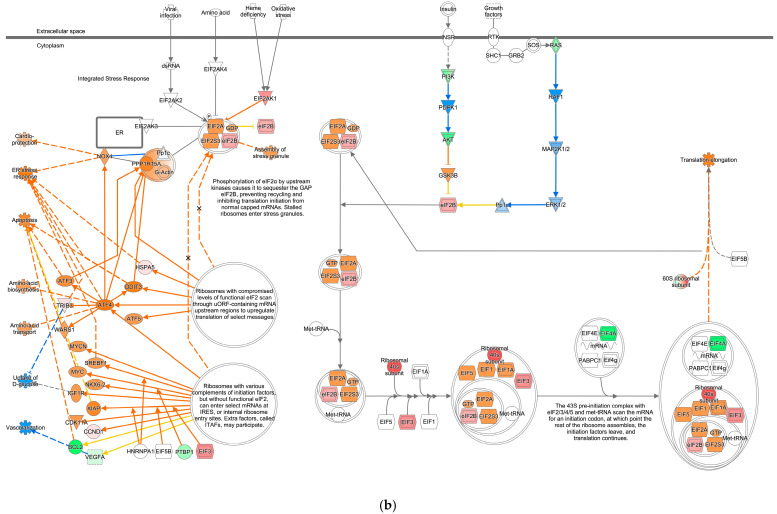
EIF2 signaling in diffuse-type and intestinal-type gastric cancer. (**a**) Computational pathway analysis suggests most of the nodes of the EIF2 signaling pathway in diffuse-type gastric cancer were predicted to be inhibited. (**b**) Computational pathway analysis suggests that most of the nodes in intestinal-type gastric cancer were predicted to be activated. Red and green indicate upregulated and downregulated gene expression, respectively. Orange and blue indicate predicted activation and inhibition, respectively. The intensities of the colors indicate the degree of up- or downregulation. Orange and blue lines indicate activation and inhibition, respectively. Solid and dashed lines indicate direct and indirect interaction, respectively.

EIF2 signaling was overlaid onto gene expression in diffuse-type and intestinal-type gastric cancer (Figure 9). Most of the nodes of the EIF2 signaling pathway in diffuse-type gastric cancer were predicted to be inhibited (Figure 9a), whereas most of the nodes in intestinal-type gastric cancer were predicted to be activated (Figure 9b) in the EIF2 signaling pathway. Translation elongation was predicted to be inhibited or activated in diffuse-type or intestinal-type gastric cancer, respectively, in computational pathway analysis. Uptake of D-glucose was predicted to be activated or inhibited in diffuse-type or intestinal-type gastric cancer, respectively. Vascularization was predicted to be inhibited in both diffuse-type and intestinal-type gastric cancer in computational pathway analysis.

### 2.8. Interaction Between EIF2 Signaling and Infection by SARS-CoV

**Figure 10:**
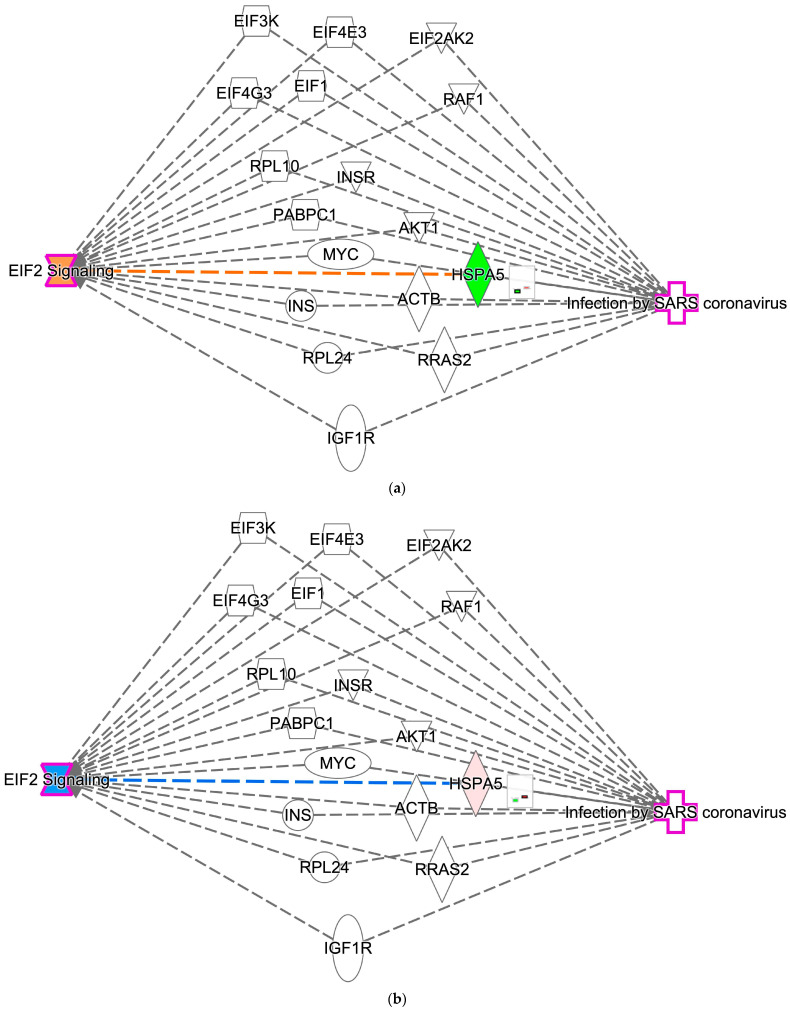
Computational analysis suggests interactions between EIF2 signaling and infection by SARS coronavirus. (**a**) The predicted interaction between EIF2 signaling and infection by SARS coronavirus in diffuse-type gastric cancer. (**b**) The predicted interaction between EIF2 signaling and infection by SARS coronavirus in intestinal-type gastric cancer. Red and green indicate upregulated and downregulated gene expression, respectively. Orange and blue indicate predicted activation and inhibition, respectively. The intensities of the colors indicate the degree of up- or downregulation. Orange and blue lines indicate activation and inhibition, respectively.

The interaction between EIF2 signaling and SARS-CoV infection was analyzed using IPA. The gene expression overlay for diffuse-type gastric cancer in relation to the interaction of EIF2 signaling and infection by SARS-CoV revealed EIF2 signaling activation and a decrease in heat shock protein family A (Hsp70) member 5 (HSPA5) in computational analysis (Figure 10a). The gene expression overlay for intestinal-type gastric cancer in relation to the interaction of EIF2 signaling and infection by SARS-CoV revealed EIF2 signaling inhibition and an increase in HSPA5 in computational analysis (Figure 10b).

The nodes that interacted with EIF2 signaling and infection by SARS-CoV included actin beta (ACTB), AKT serine/threonine kinase 1 (AKT1), eukaryotic translation initiation factor 1 (EIF1), eukaryotic translation initiation factor 2 alpha kinase 2 (EIF2AK2), eukaryotic translation initiation factor 3 subunit K (EIF3K), eukaryotic translation initiation factor 4E family member 3 (EIF4E3), eukaryotic translation initiation factor 4 gamma 3 (EIF4G3), heat shock protein family A (Hsp70) member 5 (HSPA5), insulin-like growth factor 1 receptor (IGF1R), insulin (INS), insulin receptor (INSR), MYC proto-oncogene, bHLH transcription factor (MYC), poly(A) binding protein cytoplasmic 1 (PABPC1), Raf-1 proto-oncogene, serine/threonine kinase (RAF1), ribosomal protein L10 (RPL10), ribosomal protein L24 (RPL24), and RAS-related 2 (RRAS2). Among the nodes linking EIF2 signaling and infection by SARS coronavirus, HSPA5 was identified as the gene in which gene expression was altered in diffuse-type and intestinal-type gastric cancer.

### 2.9. EIF2 Signaling in Coronavirus Replication Pathway

**Figure 11:**
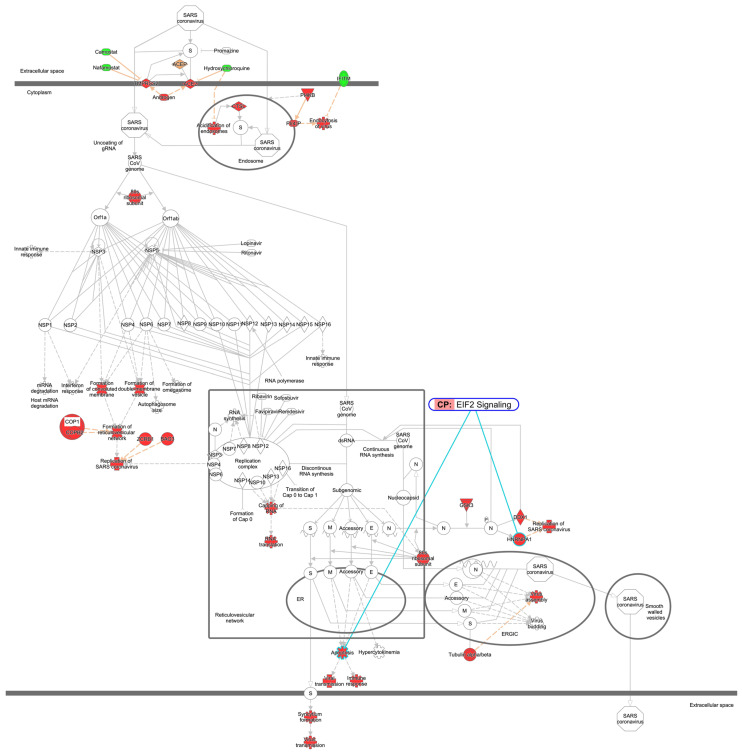
Coronavirus replication pathway and EIF2 signaling. EIF2 signaling was overlaid onto the coronavirus replication pathway in computational pathway analysis, as shown in the light blue line. The colors indicate the expected activation state of the coronavirus replication pathway. Red and green indicate upregulated and downregulated gene expression, respectively. Orange indicates predicted activation. The intensities of the colors indicate the degree of up- or downregulation. An orange line indicates activation. Solid and dot lines indicate direct and indirect interaction, respectively. CP—canonical pathway.

The EIF2 signaling and the coronavirus replication pathway are associated through heterogenous nuclear ribonucleoprotein A1 (HNRNPA1) and apoptosis (Figure 11). HNRNPA1 is a member of a family of ubiquitously expressed heterogeneous nuclear ribonucleoproteins, which are RNA-binding proteins that associate with pre-mRNAs in the nucleus and influence pre-mRNA processing, as well as other aspects of mRNA metabolism and transport.

## 3. Discussion

Molecular network analysis using the IPA network analysis tool revealed that the coronavirus pathogenesis pathway and EIF2 signaling pathway have inverse activation states. EIF2 signaling is involved in the process of protein translation. Major factors in EIF2 signaling include EIF2A, EIF2 subunit gamma (EIF2S3), eIF2B (EIF2B (complex)), and EIF2AK2. The level of phosphorylation of EIF2A is elevated in hypoxia via the induction of ER stress and PERK [7]. EIF2 signaling may be involved in various physiological conditions, including tumor progression, viral infection, amino acid starvation, heme deprivation, proteasome inhibition, and UV irradiation [7,8].

EIF2AK2 (or protein kinase double-stranded RNA-dependent; PKR) was initially identified as a kinase that phosphorylates EIF2A in response to viral infection [8]. EIF2AK2 plays important roles in eukaryotic response to viral infection, the regulation of cell growth and differentiation, apoptosis, and cancer [9]. EIF2 signaling and virus infection are closely correlated. Recent studies have demonstrated that viral infection causes an alteration in EIF2 signaling pathways [10,11,12,13,14,15]. SARS-CoV-2 infection induces beta cell transdifferentiation via the EIF2 pathway, which is mediated by PKR phosphorylation [12]. EIF2 was suggested as a potential target for reversing beta cell transdifferentiation in diabetes following SARS-CoV-2 infection [12]. Human coronavirus (HCoV) infection downregulates the EIF2 signaling pathway [10], which aligns with the findings of our study. SARS-CoV-2 nonstructural protein (NSP) 6 can trigger EIF2AK3-EIF2A pathway-mediated autophagy and inhibit interferon production [11]. Upon detection of cytoplasmic double-stranded RNA (dsRNA), mammalian cells initiate pathways including the EIF2AK2/PKR pathway, the oligo(A) synthetase–ribonuclease L (OAS-RNase L) pathway, and the RIG-I-like receptor–mitochondrial antiviral-signaling pathway [13]. Upon binding to viral dsRNA, EIF2AK2/PKR forms homodimers, which induce the autophosphorylation of Thr446 (phospho-PKR) within the activation segment of its catalytic domain and activate EIF2AK2/PKR catalytic activity [13]. EIF2AK2/PKR is a key player in the innate immune response to RNA virus infection that upregulates antiviral gene expression, including the production of interferons [16]. Coronaviruses counteract EIF2AK2/PKR-mediated signaling in order to prevent the translational shut-off due to EIF2A phosphorylation [16].

Viruses enter cells via cellular receptors and use the protein translation machinery of the infected host cells to replicate viral proteins [17,18]. Viral infection triggers cellular ISR, in which the regulation of the EIF2 pathway plays a vital role as an antiviral immune system [17]. It is likely that viral replication induces mitochondrial gene transcription and production, and mitochondrial dsRNAs, a potential major source of endogenous dsRNAs in mammalian cells, activate PKR and subsequently phosphorylate EIF2A, resulting in the inhibition of host protein translation [17,19]. The systemic inverse relationship between the coronavirus pathogenesis pathway and EIF2 signaling observed in the current study would explain why EIF2 signaling inhibition is triggered by the host antiviral immune system. It is also possible that the virus suppresses the host protein translation to promote the translation of viral proteins.

Upregulated genes in diabetes gastroenteropathy include the SARS-CoV-2 viral entry genes and genes involved in viral replication [14]. The mouse hepatitis coronavirus induces the phosphorylation of EIF2A, which leads to the formation of stress granules and processing bodies and, subsequently, host mRNA translational shut-off [20]. EIF2AK3/PERK is an ER-localized transmembrane unfolded protein response (UPR) sensor with a luminal amino-terminal domain that binds HSPA5 [15]. The UPR reduces ER load via the EIF2AK3-dependent arrest of cap-dependent translation initiation and by increasing ER-associated catabolic activities. Here, reticulophagy, a catabolic process in which ER cargo is diverted to the lysosome for degradation, plays an essential role in maintaining ER homeostasis [15]. Structural glycoprotein expression in Zaire ebolavirus is decreased during viral infection to increase the virus’s fitness via reticulophagy [21]. The expression of glycoprotein induces ER stress, which activates the UPR to stall protein translation, thereby reducing the protein load in the ER and activating the gene expression of proteins involved in protein folding [21]. EIF2AK3/PERK in the ER transmembrane activates the transcription factor ATF4 during UPR in ER stress [21].

M2 macrophages are involved in anti-inflammatory responses and allergic diseases [22,23]. The characteristics of M2 macrophages may resemble the activated state of the coronavirus pathogenesis pathway. Computational pathway analysis predicted that EIF2 signaling was activated, while the coronavirus pathogenesis pathway was inactivated in B cells. Some immune modulation may be involved, and this requires future investigation. Among the nodes linking EIF2 signaling and infection by SARS coronavirus, HSPA5 was altered in diffuse-type and intestinal-type gastric cancer. Since Hsp70 is involved in stress response, and caloric restriction increased the expression of Hsp70 in the gut [24], disease onset and malignancy might be associated with translational regulation.

There is also the possibility of knowledge base bias or circularity, with the selection of coronaviruses already known to manipulate EIF2. It is possible that EIF2 nodes are downstream of stress or viral proteins. In future investigations, orthogonal validation approaches (e.g., gene set enrichment analysis or independent dataset reanalysis) may be needed to ensure the inverse pattern is not an artifact of pathway definition.

EIF2 signaling plays a crucial role in translation initiation and beta cell transdifferentiation following severe acute respiratory syndrome coronavirus 2 (SARS-CoV-2) infection [12]. Our findings indicate that EIF2 signaling is inactivated when the coronavirus pathogenesis pathway is activated. The difference between coronavirus and SARS-CoV-2 might cause this discrepancy. In the current study, we identified multiple miRNAs (e.g., miR-378, let-7 family members, miR-34, miR-1292-3p, etc.) that interact with the EIF2 signaling pathway. An miRNA analysis in COVID-19 patients revealed that let-7 was significantly downregulated in the plasma of acute-phase COVID-19 patients [25]. Another study also demonstrated that the let-7 family was downregulated in the plasma of both mild and severe COVID-19 patients compared to healthy controls [26]. Another study demonstrated that let-7 was upregulated in the serum and urine of moderate COVID-19 cases, but not in mild or severe COVID-19 cases, compared to healthy controls [27]. These previous experimental findings indicate that some regulation in the let-7 family of miRNAs is involved in coronavirus pathogenesis. Indeed, a small-molecule C1632, which blocks the interaction between LIN28 and pri/pre-let-7, thus promoting the maturation of let-7, was reported to inhibit SARS-CoV-2 replication and virus-induced inflammation via upregulating let-7 [28]. The target genes of miRNAs identified were involved in viral signaling. 

A limitation of this study is that the interactions were analyzed in silico; thus, experimental confirmation is required. Comprehensive quantitative analyses are needed to capture the overall relationship between EIF2 signaling and coronavirus pathogenesis pathways. This could involve the following: (i) a scatter plot of EIF2 signaling vs. the coronavirus pathogenesis pathway across all analyses, including correlation coefficients and the proportion of analyses with opposite activation directions in the activity plot; (ii) measurement of EIF2 phosphorylation and translation rates in primary M2 macrophages or B cells during coronavirus infection; (iii) perturbation of selected miRNAs, such as the let-7 family, and assessment of EIF2 pathway readouts; and (iv) validation of the EIF2–coronavirus pathway interplay in gastric organoid models. The EIF2 signaling pathway is a potential target of viral infection, as viral infection and EIF2 signaling interact closely.

## 4. Materials and Methods

### 4.1. Canonical Pathway Analysis

The coronavirus pathogenesis pathway and EIF2 signaling were analyzed using the QIAGEN IPA network pathway tool [4,29] (https://digitalinsights.qiagen.com) (QIAGEN Digital Insights, Aarhus C, Denmark, and Redwood City, CA, USA) (accessed on 22 September 2025). As of 2021, 106 analyses and 106 datasets from more than 100,000 pieces of data were found to be related to SARS-CoV in the IPA database as previously described [4]. The details of activation z-scores were described in a previous study [4]. We focused on the relationship between the coronavirus pathogenesis pathway and EIF2 signaling in the current study.

### 4.2. Activity Plot Analysis

The activity plot of the coronavirus pathogenesis pathway activation z-score in the IPA database identified more than 20,000 analyses as of October 2022, which is many more than previously described [4]. Among the analyses of the coronavirus pathogenesis pathway in the IPA database, the M2 macrophage in the omentum had the highest z-score, and B cells in peripheral blood had the lowest z-score, as of October 2022.

### 4.3. miRNA Interaction Analysis

The upstream or downstream miRNA with direct interactions with the nodes in the canonical pathways was investigated. The confidence level of the miRNA was set as experimentally observed or high (predicted) in the QIAGEN IPA setting.

### 4.4. Enrichment Analysis

The enrichment analysis of the predicted target genes of the miRNAs was performed using the Database for Annotation, Visualization, and Integrated Discovery (DAVID) (https://davidbioinformatics.nih.gov/home.jsp, accessed on 15 December 2025). The predicted target genes of the miRNAs were extracted from the IPA database search. The predicted target genes of the miRNAs were then uploaded to DAVID, which resulted in the enrichment analysis of 528 *Homo sapiens* genes (9 genes were identified as unknown). Functional annotation clustering was performed using DAVID.

### 4.5. IPA Network Analysis

The analysis results for iPS cells infected with SARS-CoV-2 are available in the IPA database. The source dataset is available in the GEO public database with the GEO accession number GSE156754 (https://www.ncbi.nlm.nih.gov/geo/query/acc.cgi?acc=GSE156754, accessed on 17 December 2025) [5]. Data on intestinal-type and diffuse-type gastric cancer from The Cancer Genome Atlas (TCGA) of the cBioPortal for Cancer Genomics database (https://www.cbioportal.org, accessed on 30 September 2025) at the National Cancer Institute (NCI) Genomic Data Commons (GDC) data portal (https://portal.gdc.cancer.gov, accessed on 30 September 2025) [30,31,32,33] were uploaded and analyzed using IPA, as previously described [34,35].

### 4.6. Statistical Analysis

The RNA sequencing data on diffuse-type and intestinal-type gastric cancer were analyzed using IPA, as previously described [36]. The activation z-score in each network or pathway was calculated using IPA to show the level of activation [29].

## 5. Conclusions

The molecular network analysis revealed that the coronavirus pathogenesis pathway and EIF2 signaling frequently exhibit inverse activation states in the examined datasets. Interestingly, when the coronavirus pathogenesis pathway is activated, EIF2 signaling is inactivated, and when EIF2 signaling is activated, the coronavirus pathogenesis pathway is inactivated according to computational pathway analysis. The findings of the study may provide insight into treatments for coronavirus infection.

While the computational prediction is a valid approach for a hypothesis-generating study, experimental validation is critical to overcome inherent limitations. Future investigations—for example, validation of key protein levels, such as phosphorylated EIF2A or PKR, or testing the role of identified miRNAs in coronavirus-infected cell models—are needed to validate the computational predictions.

## Figures and Tables

**Table 1:** Basic experimental details of analysis of 1-normal-control-skin-infection-SARS-coronavirus-2-(SARS-CoV-2)-6408 (as of October 2022).

KEY	Value
GEO accession	GSE156754
Cell type	induced pluripotent stem (iPS) cell
Multiplicity of infection (MOI)	0.006 vs. mock
Time point	48 h
Sample size	4 samples (2 samples each)

**Table 2:** Summary of key EIF2 signaling genes.

Symbol	Entrez Gene Name	Log Ratio	Location	Family
ATF3	activating transcription factor 3	−0.1395	Nucleus	transcription regulator
ATF4	activating transcription factor 4	−0.471	Nucleus	transcription regulator
ATF5	activating transcription factor 5	0.344	Nucleus	transcription regulator
BCL2	BCL2 apoptosis regulator	−0.1613	Cytoplasm	transporter
CCND1	cyclin D1	1.979	Nucleus	transcription regulator
CDK11A	cyclin-dependent kinase 11A	2.2268	Nucleus	kinase
DDIT3	DNA damage inducible transcript 3	−2.0304	Nucleus	transcription regulator
EIF1	eukaryotic translation initiation factor 1	1.1765	Cytoplasm	translation regulator
EIF2A	eukaryotic translation initiation factor 2A	−1.3851	Cytoplasm	translation regulator
EIF2AK1	eukaryotic translation initiation factor 2 alpha kinase 1	1.5736	Cytoplasm	kinase
EIF2AK2	eukaryotic translation initiation factor 2 alpha kinase 2	1.6685	Cytoplasm	kinase
EIF2AK3	eukaryotic translation initiation factor 2 alpha kinase 3	1.5345	Cytoplasm	kinase
EIF2AK4	eukaryotic translation initiation factor 2 alpha kinase 4	1.3178	Cytoplasm	kinase
EIF2S3	eukaryotic translation initiation factor 2 subunit gamma	1.8189	Cytoplasm	translation regulator
EIF4E	eukaryotic translation initiation factor 4E	−0.7059	Cytoplasm	translation regulator
EIF5	eukaryotic translation initiation factor 5	0.7781	Cytoplasm	translation regulator
EIF5B	eukaryotic translation initiation factor 5B	1.9156	Cytoplasm	translation regulator
GRB2	growth factor receptor-bound protein 2	0.1087	Cytoplasm	other
GSK3B	glycogen synthase kinase 3 beta	1.1651	Nucleus	kinase
HNRNPA1	heterogeneous nuclear ribonucleoprotein A1	2.5084	Nucleus	other
HSPA5	heat shock protein family A (Hsp70) member 5	1.4574	Cytoplasm	enzyme
IGF1R	insulin-like growth factor 1 receptor	2.551	Plasma Membrane	transmembrane receptor
INSR	insulin receptor	1.7649	Plasma Membrane	kinase
MYC	MYC proto-oncogene, bHLH transcription factor	0.5141	Nucleus	transcription regulator
MYCN	MYCN proto-oncogene, bHLH transcription factor	1.4211	Nucleus	transcription regulator
NKX6-2	NK6 homeobox 2	−0.3426	Nucleus	transcription regulator
NOX4	NADPH oxidase 4	3.2769	Cytoplasm	enzyme
PABPC1	poly(A) binding protein cytoplasmic 1	2.2335	Cytoplasm	translation regulator
PDPK1	3-phosphoinositide-dependent protein kinase 1	1.0673	Cytoplasm	kinase
PPP1R15A	protein phosphatase 1 regulatory subunit 15A	0.5765	Cytoplasm	other
PTBP1	polypyrimidine tract binding protein 1	1.947	Nucleus	enzyme
RAF1	Raf-1 proto-oncogene, serine/threonine kinase	1.5109	Cytoplasm	kinase
SHC1	SHC adaptor protein 1	0.9844	Cytoplasm	other
SREBF1	sterol regulatory element binding transcription factor 1	0.7003	Nucleus	transcription regulator
TRIB3	tribbles pseudokinase 3	1.7107	Nucleus	kinase
VEGFA	vascular endothelial growth factor A	−0.0891	Extracellular Space	growth factor
WARS1	tryptophanyl-tRNA synthetase 1	1.48	Cytoplasm	enzyme
XIAP	X-linked inhibitor of apoptosis	0.1357	Cytoplasm	enzyme

**Table 3:** The microRNAs (miRNAs) and the group that have direct relationships with EIF2 signaling.

microRNAs (miRNAs) and Group
let-7
miR-1292-3p (miRNAs w/seed CGCGCCC)
miR-15
miR-34
miR-378
miR-493
miR-497
miR-7
miR-8
MIRLET7

**Table 4:** Predicted target genes of the microRNAs (miRNAs) and the group that have direct relationships with EIF2 signaling.

miRNAs and Group	Targets
let-7	ACVR1C, ADRB1, ANKH, APC, APC2, BCAP29, BCL2L1, BMPR1A, BTG2, CASP3, CCND1, CEBPD, CHRNA7, CPEB3, CPSF1, DDX18, DNAJB9, DORIP1, EDEM1, EDEM3, EIF4A1, ERO1A, EZH2, FN1, GAB2, GALE, GREB1, HMGA2, IGF1R, IL10, IRS2, ITGB3, KRAS, LIN28A, LRIG1, LSM6, MT-ND4, MYC, NR2E1, NRAS, PABPC4, PBX2, PNKD, PPP1R12B, RAS, RBM38, SLC1A4, SMAD2, SMAD4, STARD13, STAT3, STYK1, TARBP2, TGFBR1, TLR4, TMPRSS2, TRIB1, ZC3H3, ZNF512B
miR-1292-3p (miRNAs w/seed CGCGCCC)	AARD, ABHD17A, ACKR2, ACSL6, ADAMTS17, ANKRD33B, ANKRD62, AP5Z1, APOBEC3A, APOL6, ARHGAP1, ARHGAP17, ARHGAP19-SLIT1, ARID3A, ARL5C, ARSK, ASB10, ASL, ATAD3C, B3GNT4, BORCS5, BORCS7, BPNT1, BRSK2, BTBD19, C10orf55, C10orf95, C16orf92, C1orf174, C1QL2, C1QTNF4, C2CD4C, C8orf44-SGK3, C8orf82, C9orf163, CACNA1C, CACNG2, CACNG8, CAPN6, CASKIN1, CASP16P, CASS4, CBARP, CBLN1, CBY3, CCDC137, CCDC71, CCND1, CD209, CD82, CDH4, CDKL1, CDKN2AIPNL, CDKN2B, CEND1, CERS1, CFAP418, CFAP92, CHRNB1, CKAP4, CNNM1, CPLX1, CPSF4, CRK, CRTC1, CRYAA, CSNK1G2, CSRNP1, CTD_2207O2312, CTF1, CYB561, CYB5D2, CYP1A2, DAND5, DAZAP1, DFFB, DGCR8, DISP2, DMPK, DNAJC28, DOCK8-AS1, DOK7, DPP9, DSG3, DUOXA2, EMILIN1, EML3, ENPP7, ERAP2, ERN1, ESCO2, EVL, EXTL1, FAM89B, FBXL18, FBXL8, FHDC1, FKRP, FOXD1, G0S2, GAS6, GATD1, GFER, GMPS, GNA11, GRB2, GRB7, GRM6, GSC, GTF2H2C, HAGHL, HCN2, HEPACAM, HES2, HES5, HES7, HIC2, HMG20B, HMX2, HMX3, HOXC6, IER2, IL10, IL17RE, ING5, IRX2-DT, ISLR2, ITGAL, JAKMIP3, JUND, KBTBD6, KCNA7, KCND3, KCNE5, KCNK3, KCNMB1, KIAA0319L, KIAA0753, KLF2, KLHDC4, KLHL17, KPNA6, KREMEN1, LAIR1, LCNL1, LILRB3, LINC01124, LINC01521, LINC01551, LLPH, LMLN, LMNTD2, LOC283731, LPCAT1, LRRC14, LRRC2, LRRC45, LRRN4CL, LSM4, LTO1, LYRM2, MACC1, MAFA, MAFB, MAFF, MAFK, MAPK4, MATN4, MCM4, MELTF, MEX3D, MGAT4B, MID1IP1, MIF-AS1, MINAR2, MLANA, MLYCD, MMP25, MNX1, MRTO4, MS4A7, MSRA, MT-ATP6, NAT14, NCBP3, NCR1, NDE1, NEUROD2, NFIC, NKX2-5, NKX6-2, NKX6-3, NOTO, NRARP, NRDE2, NRXN2, NTN1, NXN, OLFML2A, OLIG1, ONECUT3, OPRD1, OR51E2, OSCAR, OTUD6A, OTX1, PAX2, PAXIP1-AS2, PCDH1, PEX6, PGPEP1, PHLDB3, PITX1, PLEKHB2, PLPPR3, POLR2D, POLR2J2/POLR2J3, POU2F2, POU3F1, PPEF2, PPP1R3B, PPP1R3G, PPT1, PRDM12, PRKN, PRR23A, PSMD5, PTBP1, RAB22A, RAB3B, RAB40C, RASL10A, RAX, RDH13, RNF223, RP11_1148L69, RP11-644F5.10, RPL23, RPL3L, RPL7L1, RPRML, RPS29, RXFP3, SAPCD2, SCIN, SERPINF2, SGK3, SHH, SHOX, SHROOM1, SKIDA1, SLC11A1, SLC12A5, SLC16A4, SLC35E2B, SLC38A12, SLC50A1, SLC52A3, SLC75A1, SLC9A7, SMIM22, SNORC, SOX12, SOX21, SP140L, SP9, SREBF1, SSTR1, SSU72-AS1, TAF13, TBC1D26, TBCCD1, TCEANC2, TEF, TEX22, TICAM1, TLX2, TLX3, TMEM107, TMEM121, TMEM130, TMEM204, TMEM250, TMEM41A, TMEM41B, TNFAIP8L1, TNNC2, TOM1, TPGS1, TRIM72, TSPAN11, TSPAN17, TSPYL1, TTLL7, TUFT1, UCKL1, UNC13A, UPF1, UPK3BL1, URM1, VAV2, VIPR1, VMO1, VSIG8, WDR81, WDR82, WIPI1, WNT7B, YME1L1, ZBTB3, ZBTB46, ZIC4, ZMAT3, ZNF101, ZNF275, ZNF282, ZNF490, ZNF514, ZNF556, ZNF561, ZNF606-AS1, ZNF682, ZNF696, ZNF771, ZNF777, ZNF850-DT
miR-15	APP, AR, BACE1, BCL2, BCL2L2, BTRC, CCND1, CCND3, CCNE1, CDC25A, CDK4, CDK6, CHEK1, EIF2B2, EIF4G2, FASN, FGF2, IFNG, MAPK3, MCL1, MYB, PPARG, PPM1D, PTGS2, PURA, RAF1, RECK, RNF125, UCP2, WEE1, WNT3A
miR-34	AR, BCL2, BIRC3, CACNB1, CCND1, CCND2, CCNE1, CCNE2, CD47, CDK4, CDK4/6, CDK6, CNTN2, CPLX2, CREB1, CSF1R, CTNND2, DNM1L, E2F3, E2F5, EFNB1, EMP1, FAM76A, GFRA3, GRM7, KCNH2, LEF1, LIN28A, MDM4, MET, MYB, MYC, MYCN, NOTCH1, NOTCH2, PPP1R10, REM2, RUNX2, SEMA4B, SHPK, SIRT1, SLC30A3, STIM1, STMN1, TP53
miR-378	CASP9, GRB2, MEG3, PDPK1, SUFU, TUSC2
miR-493	IGF1R
miR-497	BTRC, CCND1, CCND3, CCNE1, CDC25A, CDK4, MAPK3, RAF1
miR-7	ABCC1, EGFR, IDE, IGF1R, INSR, IRS2, PIK3R3, RELA, SCAP, SMAD2
miR-8	ACTN1, ADAM12, AMOTL2, ARHGAP5, BMI1, CCND1, CCND2, CCNE2, CCNG2, CDC25A, CDC27, CDC42, CDK6, CFL2, CLDN1, CLDN12, CLDN23, CRK, CRKL, CRTAP, CTNNB1, DNAJC3, E2F3, EGFR, EGR2, ERRFI1, ETS1, FAT1, FAT2, FGFR2, FHOD1, FOXF2, FTH1, GOLIM4, GSE1, HOTAIR, ITGAV, ITGB6, JAZF1, LATS1, LATS2, MAP3K1, MAPK12, MCM4, MET, MSN, MYC, MYH10, NECTIN2, OCLN, PAK6, PDE1A, PGR, PIP5K1A, PLAG1, PTK2, PTPN13, PTPN14, ROCK2, RPS6KB1, SMAD2, SMAD5, SNAI1, SNAI2, SOX2, SOX2-OT, STAT4, STAT5A, TGFB2, TGFBR1, TIAM1, TJP1, TLN1, TOB1, TP53, WASL, XBP1, XIAP, YAP1, YES1, YWHAB, YWHAG, YWHAZ, ZEB1, ZEB2, ZFP36
MIRLET7	COL1A1, COL1A2, COL4A5, COL5A2, HMGA2, KRAS, MYC, RAS

**Table 5:** Functional annotation clustering of the enrichment analysis of predicted target genes in the Database for Annotation, Visualization, and Integrated Discovery (DAVID) (clusters 1 to 4).

Cluster	Cluster Enrichment Score	Category	Term	*p*-Value
1	11.51	KEGG_PATHWAY	Chronic myeloid leukemia	2.07 × 10^−13^
1	11.51	KEGG_PATHWAY	Hepatitis B	2.2 × 10^−13^
1	11.51	KEGG_PATHWAY	Cellular senescence	3.99 × 10^−12^
1	11.51	KEGG_PATHWAY	Human T-cell leukemia virus 1 infection	4.26 × 10^−12^
1	11.51	KEGG_PATHWAY	Viral carcinogenesis	3.54 × 10^−10^
2	10.45	GOTERM_BP_DIRECT	Positive regulation of transcription by RNA polymerase II	1.85 × 10^−19^
2	10.45	GOTERM_CC_DIRECT	Chromatin	7.66 × 10^−18^
2	10.45	GOTERM_MF_DIRECT	DNA-binding transcription factor activity, RNA polymerase II-specific	2.61 × 10^−16^
2	10.45	GOTERM_MF_DIRECT	DNA-binding transcription activator activity, RNA polymerase II-specific	3.48 × 10^−14^
2	10.45	GOTERM_MF_DIRECT	Sequence-specific double-stranded DNA binding	7.26 × 10^−14^
2	10.45	GOTERM_MF_DIRECT	RNA polymerase II cis-regulatory region sequence-specific DNA binding	1.23 × 10^−13^
2	10.45	GOTERM_MF_DIRECT	DNA-binding transcription factor activity	3.84 × 10^−12^
2	10.45	GOTERM_CC_DIRECT	Nucleus	4.86 × 10^−11^
2	10.45	GOTERM_BP_DIRECT	Regulation of transcription by RNA polymerase II	6.46 × 10^−10^
2	10.45	UP_KW_MOLECULAR_FUNCTION	Activator	1.11 × 10^−9^
2	10.45	GOTERM_CC_DIRECT	Nucleoplasm	1.81 × 10^−7^
2	10.45	UP_KW_BIOLOGICAL_PROCESS	Transcription regulation	3.37 × 10^−7^
2	10.45	UP_KW_MOLECULAR_FUNCTION	DNA binding	5.06 × 10^−7^
2	10.45	UP_KW_BIOLOGICAL_PROCESS	Transcription	7.94 × 10^−7^
2	10.45	UP_KW_CELLULAR_COMPONENT	Nucleus	9.58 × 10^−7^
2	10.45	GOTERM_MF_DIRECT	DNA binding	1.54 × 10^−5^
3	7.44	KEGG_PATHWAY	p53 signaling pathway	1.47 × 10^−10^
3	7.44	GOTERM_BP_DIRECT	G1/S transition of the mitotic cell cycle	1.05 × 10^−9^
3	7.44	KEGG_PATHWAY	Epstein–Barr virus infection	3.06 × 10^−4^
4	7.29	KEGG_PATHWAY	Small-cell lung cancer	1.01 × 10^−12^
4	7.29	KEGG_PATHWAY	Measles	4.47 × 10^−7^
4	7.29	KEGG_PATHWAY	Epstein–Barr virus infection	3.06 × 10^−4^

**Table 6:** Nodes in the coronavirus pathogenesis pathway overlapped with EIF2 signaling.

Nodes
43S TRANSLATION PREINITIATION
48s
60S ribosomal subunit
Eif2
EIF4F
Met-tRNA-eIF2
PI3K
Apoptosis
ATF4
BCL2
CCND1
DDIT3
EIF2A
EIF2AK3
EIF4E
ERK1/2
Ribosomal 40s subunit

## Data Availability

Dataset available on request from the authors.

## Supplementary Materials

The following supporting information can be downloaded at https://www.mdpi.com/article/10.3390/ijms27031525/s1.

## Abbreviations

The following abbreviations are used in this manuscript:

EIF2	Eukaryotic initiation factor 2
miRNA	microRNA
GDP	Guanosine 5′-diphosphate
Met-tRNAi	Initiator tRNA
GTP	Guanosine 5′-triphosphate
ISR	Integrated stress response
HRI	Heme-regulated inhibitor
EIF2AK	EIF2 alpha kinase
PKR	Double-stranded RNA-dependent protein kinase
PERK	PKR-like endoplasmic reticulum kinase
GCN	General control non-repressible
MAPK	Mitogen-activated protein kinase
IFN	Interferon
TGF	Transforming growth factor
NF	Nuclear factor
IPA	Ingenuity Pathway Analysis
SARS-CoV	Severe acute respiratory syndrome coronavirus
iPS	Induced pluripotent stem
eIF2B	EIF2B (complex)
GEO	Gene Expression Omnibus
GAIT	Gamma-activated inhibitor of translation
MOI	Multiplicity of infection
DAVID	The Database for Annotation, Visualization, and Integrated Discovery
HSPA5	Heat shock protein family A (Hsp70) member 5
EIF2S3	EIF2 subunit gamma
HCoV	Human coronavirus
NSP	Nonstructural protein
dsRNA	Double-stranded RNA
OAS-RNase L	Oligo(A) synthetase–ribonuclease L
UPR	Unfolded protein response
TCGA	The Cancer Genome Atlas
NCI	National Cancer Institute
GDC	Genomic Data Commons
